# Draft Genome Sequence of Pediococcus pentosaceus Strain PP16CC, Isolated from Oyster Crassostrea corteziensis

**DOI:** 10.1128/mra.00395-22

**Published:** 2022-09-13

**Authors:** Julio A. Hernandez-Gonzalez, Ricardo Vazquez-Juarez, Jose Manuel Vazquez-Guillen, Carlos Rangel-Davalos, Cristina Rodriguez-Padilla, Maurilia Rojas

**Affiliations:** a Laboratorio de Ciencia y Tecnología de los Alimentos, Universidad Autónoma de Baja California Sur, La Paz, B.C.S., Mexico; b Laboratorio de Genómica y Bioinformática, Centro de Investigaciones Biológicas del Noroeste, La Paz, B.C.S., Mexico; c Laboratorio de Inmunología y Virología, Facultad de Ciencias Biológicas, Universidad Autónoma de Nuevo León, Monterrey, Nuevo León, Mexico; Indiana University, Bloomington

## Abstract

Pediococcus pentosaceus strain PP16CC comes from the intestine of Crassostrea corteziensis. A 1.82-Mbp draft genome of this strain was assembled using A5-miseq from illumina reads, resulting in 4 contigs and 1,856 predicted protein coding genes. Additionally, 23 proteins belonging to various glycosyl hydrolase families and 6 prophage regions were identified.

## ANNOUNCEMENT

Crassostrea corteziensis is a potential alternative in oyster farming of Mexican Pacific (1). However, a main problem has been diseases (2); therefore, probiotic Pediococcus pentosaceus has been studied to improve oyster survival during seed production (3).

P. pentosaceus belongs to the *Lactobacillaceae* family, and in a core genome phylogenetic analysis of this family, it forms a monophyletic clade with the heterofermentative Lactobacillus plantarum group and the pediococci (4). The pangenome and core genome of P. pentosaceus have 7,938 and 1,240 genes, respectively, and the main differences between strains were found in carbohydrate metabolism and horizontally transferred DNA (5).

P. pentosaceus strain PP16CC was isolated on May 2011 using MRS agar at room temperature in anaerobiosis from *C. corteziensis* harvested from the Pacific Ocean and preserved with glycerol at −85°C. DNA of the strain was extracted using the Wizard genomic DNA purification kit (Promega, USA). The quality and quantity of DNA were determined via the Quant-iT PicoGreen double-stranded DNA (dsDNA) assay kit on the Qubit 2.0 fluorometer (both from Thermo Fisher Scientific, USA). The DNA library was prepared using the Nextera DNA Flex library prep kit (Illumina) and sequenced with the MiSeq reagent kit v2 (300 cycles), yielding 627,419 paired-end reads with an average length of 151 bp. Reads were quality filtered and assembled with the A5-miseq pipeline v. 20160825 (6) and SSPACE v. 3.0 (7), resulting in a draft genome with a G+C content of 37.0235%, a total length of 1,820,443 bp in 4 contigs (*N*_50_, 527,937 bp), and an average coverage of 84-fold.

Taxonomy was established by the Microbial Genome Atlas (MiGA) (8), determined that the PP16CC strain belongs to the genus *Pediococcus* (*P* = 0.00153) and to the species *P. pentosaceus* (*P* = 0.0198). And its closest relatives are *P. pentosaceus* GCA_004354495.1 (98.86% average nucleotide identity [ANI]) and *P. pentosaceus* GCA_001437285.1 (98.83% ANI).

The contig order was obtained via mauve contig mover (9) using the genome of P. pentosaceus ATCC 25745 as a reference (NC_008525.1). The genome annotation was performed using PGAP v. 6.0 (10) and predicted 1,794 coding sequences, 7 rRNA genes, 52 tRNA genes, and 3 noncoding RNA genes.

Glycosyl hydrolase (GH) enzymes were annotated with the dbCAN metaserver (11, 12) using the carbohydrate-active-enzyme database (13), identifying 23 proteins belonging to GH families, of which some were repeated. Seven of eight families that Jiang et al. (5) found in most P. pentosaceus genomes were GH1 (3.2.1.86), GH25 (3.2.1.17), GH73 (Unspecified NA), GH65 (2.4.1.8), GH2 (3.2.1.23), GH126 (NA), and GH13_29 (3.2.1.93); in addition, 5 families were identified, namely, GH31 (3.2.1.177), GH78 (3.2.1.40), GH43_26 (3.2.1.-), GH23 (NA), and GH170 (NA).

Integrated prophages were predicted using PHASTER (14), six prophage regions, three intact and three incomplete were identified (Table 1). The phages with the highest number of proteins (values in parenthesis, Table 1) similar to prophage regions 2, 4, and 6 were *Listeria* virus LP101, *Lactococcus* phage P335 *sensu lato*, and *Lactobacillus* phage phig1e, respectively. All three belong to the *Siphoviridae* family. Software packages were used with default parameters.

**TABLE 1:** General characteristics of the prophage region

Region	Region length (Kbp)	Completeness	Score	Total no. of proteins	Region position	Accession no. for the most common phage
1	14.8	Incomplete	20	20	Scaffold2 102192–117013	NC_031036 (3)
2	25.9	Intact	130	31	Scaffold2 122233–148190	NC_024387 (11)
3	29	Incomplete	40	12	Scaffold2 284517–313522	NC_023719 (2)
4	26.5	Intact	150	35	Scaffold2 425474–452037	NC_004746 (6)
5	24.1	Incomplete	30	21	Scaffold2 452054–476230	NC_019489 (2)
6	51.8	Intact	150	75	Scaffold3 236382–288269	NC_004305 (14)

### Data availability.

The whole-genome shotgun project for P. pentosaceus PP16CC was deposited at DDBJ/ENA/GenBank (JALCZR000000000) and under BioProject number PRJNA814659, BioSample number SAMN26563464, and SRA SRR18292892.
